# Media Device Use and Vision Disorders in the Pediatric Age: The State of the Art

**DOI:** 10.3390/children11111408

**Published:** 2024-11-20

**Authors:** Elena Bozzola, Mariangela Irrera, Romie Hellmann, Salvatore Crugliano, Michele Fortunato

**Affiliations:** 1 Pediatric Unit, Bambino Gesù Children’s Hospital, IRCCS, 00100 Rome, Italy; mariangela.irrera@opbg.net; 2 Faculty of Medicine, Saint Camillus International University of Health Sciences, 00100 Rome, Italy; u.008376@students.unicamillus.org; 3 ASST Valleolona, 21052 Busto Arsizio, Italy; salvatore.crugliano@asst-valleolona.it; 4 Association Internationale pour l’Enfance et la Réhabilitation Visuelle, 75016 Paris, France; michele.fortunato@opbg.net; 5 Italian Association of Pediatric Ophthalmologists and Paediatricians, 00100 Rome, Italy

**Keywords:** media device, eye, children, adolescents, health

## Abstract

Introduction. Evidence is consistent with increased screen viewing time among children and adolescents, and anticipation at the age at which children interact with media devices. Incorrect use of technology, as well as overuse, may lead to serious consequences. This study aims to revise scientific international literature and to describe the potential eye risks correlated to screen viewing time in the pediatric age. Materials and Methods. A review of the literature was performed according to the PRISMA 2020 guidelines, using the search terms “media device” and “eye” with the filter “age 0–18”. Results. Analyzing the international literature, we found 26 articles. Pre-myopia, myopia, digital eye strain, and acute acquired comitant esotropia were listed as potential and time-related consequences associated with the incorrect use of media devices among children and adolescents. Discussion. Family education on media device exposure and potential risk for children and adolescents’ sight in case of prolonged digital/screen exposure is required. During pediatric check controls, a dialogue with families on prolonged media device use at a close distance should be undertaken. Pediatricians and ophthalmologists should consider screen viewing time in case of ocular problems.

## 1. Introduction

Screen viewing time (SVT), or digital/screen exposure, is the total time spent by an individual viewing or using any digital or electronic device, such as televisions, smartphones, tablets, videogames, or computers [1].

Evidence is consistent with children starting to use online platforms at an early age in industrialized countries [1,2,3]. In America, a study showed that 96.6% of young children aged 6 months to 4 years used mobile devices, and most began to use them before the age of 1 year [2]. Evidence suggests a constant use of electronic devices, with even 68% of children under age 3 using screen media daily [3].

According to the American Academy of Pediatrics guidelines, children younger than 24 months should not be exposed to media devices and those aged 2 to 5 years should keep their SVT to under 1 h/day [4].

Young Italian children are overly exposed to mobile devices. The Italian Paediatric Society recommends no media device exposure in children aged less than 2 years, no more than 2 h daily in those aged 2–5 years, and no more than 4 h/day in older children [5].

Nevertheless, media device use in early childhood is still a matter of concern because of the potential risks. According to the literature, unhealthy and excessive media device exposure may lead to physical and neuropsychological impairments, including depression, hostile and aggressive behavior, obesity, musculoskeletal discomfort, and sleep and eye disorders [6,7,8,9]. As well as in early childhood, in adolescence prolonged media device use may negatively influence the psychophysical development of the adolescent, such as in learning, sleep, and sight. Moreover, obesity, distraction, addiction, cyberbullying, and Hikikomori phenomena are described in adolescents who use media devices too frequently. The Italian Paediatric Society provides action-oriented recommendations for families and clinicians to avoid negative outcomes [10].

This study aims to examine international awareness of the impact of media device exposure on the eye in childhood.

## 2. Material and Methods

A review of the literature was performed according to the PRISMA guidelines [11] on 30 May 2024. An electronic search was undertaken, first using the PubMed database, searching for “media device” and “eye” with the filter “age 0–18”, article language “English”, and publication date of the last ten years. Then, we investigated other database sets, namely Embase, Web of Science, and Scopus. On Embase we used the search terms “media device” AND “eye”, from 2014 to 2024; on Web of Science, the topics “media device” and “eye” were refined by publication years from 2014 to 2024, and limited to infant or child preschool or child or adolescent; and on Scopus, the keywords “media device” and “eye” were limited to adolescent or child or infant, with publication date from 2014 to 2024.

Duplicates were identified and unnecessary copies were excluded. Authors independently evaluated titles and abstracts produced by the literature analysis to limit bias and mistakes. Studies were considered eligible if they met the following inclusion criteria:

- full-length articles or reviews;
- on children and adolescents up to 18 years old;
- reports dealing with media device consequences on the eye;
- English language.

The exclusion criteria were:

- reports including adults (>18 years);
- reports dealing with other themes.

Then, full texts were evaluated for eligibility by the authors. If full-text articles could not be found, an attempt to contact authors was performed to obtain the full text.

According to the PRISMA guidelines, articles not included in the original PubMed search but considered relevant to the report were evaluated. All the authors conducted a 1 h discussion, examining doubts regarding inclusion/exclusion for any report.

Additionally, to ensure further data were not missed, a second search was undertaken in the PubMed database using the keywords “media device” and “myopia”, “media device” and “digital eye strain”, and “media device” and “acute acquired comitant esotropia”. The same filters of the previous search were used.

## 3. Results

**Figure 1:**
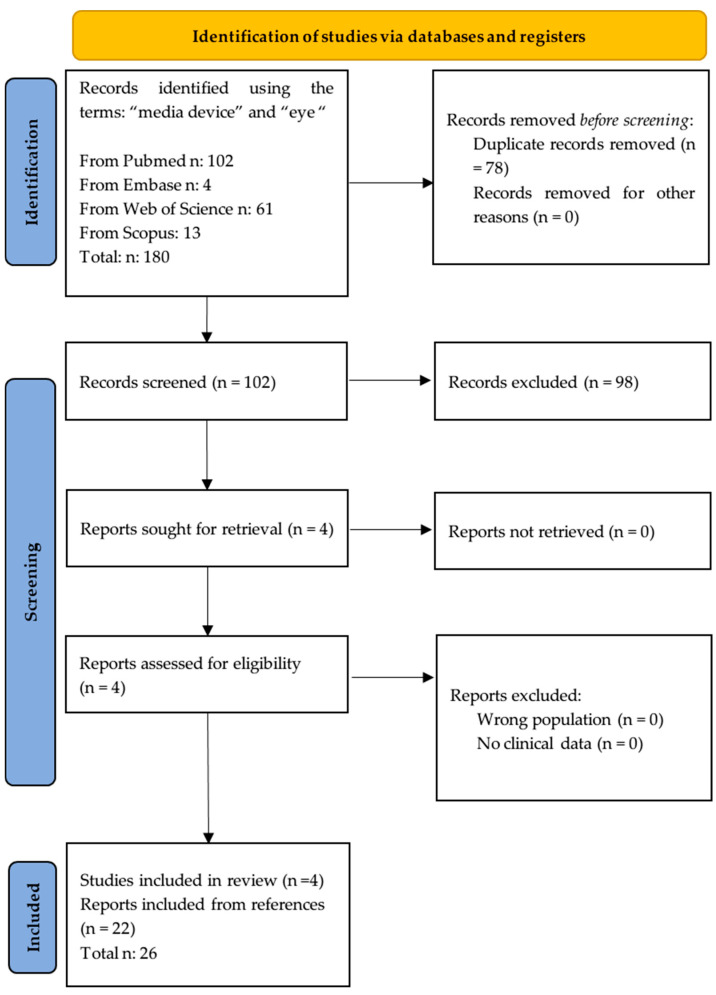
Flow chart of the selected process.

From PubMed, out of the 102 examined papers, only 4 papers fulfilled the criteria for inclusion in the review [12,13,14,15]. As detailed in Figure 1, 98 articles were excluded because they dealt with other themes (94), involved adults (3), or did not provide supporting data (1). From the references of the examined papers, 22 articles were selected and added to the study [16,17,18,19,20,21,22,23,24,25,26,27,28,29,30,31,32,33,34,35,36,37]. Regarding the other databases, according to our search strategy and inclusion criteria, we found 4 results in Embase, 61 in Web of Science, and 13 in Scopus. Nevertheless, we did not include them in the review because they were all duplicates of the PubMed results.

Analyzing the manuscripts, myopia, digital eye strain, and acute acquired comitant esotropia were the most frequent consequences reported by the literature.

From the second search, we found six results pertaining to myopia, four to digital eye strain, and zero to acute acquired comitant esotropia. Of these, four were already included, one involved either adults or children, and five dealt with other themes [12,13,14,15].

### 3.1. Myopia

From the literature search, 18 reports dealt with the risk of pre-myopia and myopia in childhood and adolescence linked to media device exposure [12,14,15,16,17,18,19,20,21,22,23,24,25,26,27,28,29,30]. Table 1 summarizes the evidence. The evidence suggests that the risk is mainly time-related [14,16,17,18,19]. Either in childhood or in adolescence, a high use of screen devices is associated with the risk of developing myopia, defined as “a condition in which the spherical equivalent refractive error of an eye is ≤−0.5 D when ocular accommodation is relaxed” [12,17,30,38]. Many authors reported media devices as harmful and gave objective proof referring to different types of devices. Many authors reported smartphones and computers being more harmful. In detail, a more myopic spherical equivalent refraction (SER) and a longer axial length (AL) were associated with more time on smartphones and computers, but not on tablets and television [20,21,22]. Computers and smartphones were also linked with an increased risk of myopia compared to television [23]. Indian reports also correlated refractive deficit with television [24,25]. Behavioral and modifiable risk factors regarding myopia were evaluated in scientific reports [12,15,16,18,21,24,25]. In detail, reduced physical activity and Westernized dietary habits have been associated with media devices [12,15,16,18,21,24,25]. A high socio-economic status has also been discussed as a potential risk factor, as students attending private schools were more likely to have access to technology [24].

Of note, screen exposure in the postnatal first year, which may be a sensitive period, may lead to later development of myopia in the preschool age [26].

Either in childhood or in adolescence, the high use of a screen device is associated with the risk of developing myopia [12,17]. Childhood and adolescent vision health declined during the COVID-19 period, likely due to increased SVT and online e-learning, an inevitable consequence of lockdown and restrictive measures. In this period, significant increases in myopia and myopic progression were reported [12,15,23,27,28]. The mean number and time of online classes, as well as the total digital screen exposure duration, were negatively correlated with myopia [29].

#### 3.1.1. DES (Digital Eye Strain)

According to the search strategy, seven reports dealt with the risk of digital eye strain (DES) in childhood linked to media device exposure, as reported in Table 2 [13,31,32,33,34,35,36]. In the literature, DES or computer vision syndrome is the term used to collectively indicate “dry eyes, impaired vision, near-sightedness, headaches, and eye fatigue” attributed to prolonged usage of desktops, laptops, mobile phones, etc. The main symptoms include blurring, redness, visual disturbance, secretion, inflammation, lacrimation, and dryness. [31]

Its pathophysiology is multifactorial, with several contributing factors, including reduced contrast level of letters compared to the background of digital screens, screen glare and reflections, wrong distance and angle of viewing digital screens, poor lighting conditions, improper posture during usage, and infrequent blinking of eyes [31,32]. A reduced blink rate and faster evaporation of the tear film in the case of prolonged media device use may be indicative of DES. [31] Additionally, the eye focusing and ocular movements required for better visibility of digital screens place additional demand on an intricate balance between accommodation and convergence mechanisms, thus making people with uncorrected or under-corrected refractive errors increasingly susceptible.

The most used devices, such as mobile phones and smartphones, are consulted within a proximal distance from the eyes. It has been suggested that smartphones affect distance visual acuity when used at a close distance, while there was no significant evidence regarding computer use [31,32]. After a few weeks wash out from mobile devices, both subjective symptoms and objective signs of the ocular impairment may improve and even disappear [31]. Outdoor activity as well as living in a rural context have been postulated to represent protective factors in either childhood and adolescence [31,32].

Compared to conventional light sources, such as incandescent and fluorescent bulbs, computer displays and smartphone screens emit a higher proportion of blue light (380–500 nm), which is associated with DES symptoms. Evidence suggests that longer smartphone use per day can increase the prevalence rates and odds of risk of ocular symptoms, as well as the likelihood of having multiple ocular symptoms [31,32]. Multivariate analysis revealed that smartphone use, prolonged use of devices, and mobile games are independent risk factors for DES in children [31,33,34,35]. Smartphones are the most frequently linked to ocular symptoms but evidence is increasing also regarding video games. This might be explained by the increased mechanical compression exerted by the contracted eyelid muscles during the child’s concentration effort on their plastic cornea [36]. The effect was particularly of note during the COVID-19 era, due to the prolonged use of media devices [33,34,35].

#### 3.1.2. Acute Acquired Comitant Esotropia

Acute acquired comitant esotropia (AACE) may be defined as an unusual manifestation of esotropia in older children without limitation of eye movement. Although the etiology is still debated, evidence suggests a possible relationship between AACE and excessive smartphone use, as reported in Table 3. The main identified risk factors were the prolonged time spent on media devices and the close reading distance. Authors assumed that excessive near work causes abnormalities in the balance between convergence and divergence, which increases the tonus in the medial rectus muscle, resulting in the development of esotropia [35,37]. COVID-19 lockdown with school closure and home confinement impacted lifestyle behavior in the young population, including a significant increase in screen time and an increase in the risk of AACE [35].

#### 3.1.3. Data Analysis According to Group Age

Considering children aged less than 12 years, and adolescents aged 13 to 18 years, 12 reports were analyzed [16,17,18,19,21,22,26,28,31,32,36]. Regarding the others, data referred to the pediatric population but without a clear distinction of the results from childhood to adolescence.

In detail, nine studies focused on childhood [16,18,19,21,22,26,28,31,36]. A prolonged screen vision is associated with the risk of developing eye impairment, myopia, and dry eye syndrome [18]. The duration of computer, smartphone, and video game screen use correlates to irritation, burning sensation, decreased visual acuity, myopia, and dry eye syndrome in several studies [18,21,31,36]. Analyzing risk factors correlated with myopic refractive errors, computers and smartphones but not television have been identified in childhood [21,22]. The first postnatal year represents a sensitive period, as screen exposure in early life can be significantly and positively associated with myopia in childhood [18,19,26]. Myopia prevalence increased with daily exposure duration and total years of exposure [16,19,26]. Screen time exposure correlates with increased myopic spherical equivalent refraction, shorter corneal radius, and higher axial length/corneal radius [19]. Children spending 3 or more hours daily had a fivefold prevalence of eye impairment [16]. Myopia prevalence increased in the COVID-19 era, likely due to more screen time [12,28].

Out of the included articles, three present results focused on adolescence [16,17,32]. Adolescents with a high exposure to smartphones were found to have higher prevalence rates for ocular symptoms, including inflammation, redness, and lacrimation, and for myopia [16,17,32]. Prolonged daily smartphone use links with a higher risk of multiple ocular symptoms [32]. It is a matter of speculation whether the increasing prevalence of eye impairment is related to light exposure, alteration in circadian rhythms, or reduced outdoor activity. Evidence suggests both low physical activity and prolonged screen device exposure are significant contributors to sight problems [17].

Compared to the pre-pandemic period, in the COVID-19 era, the mean duration of the digital use increased. Multivariate analysis revealed smartphone use, and prolonged use of devices and mobile games, are independent risk factors for dry eye syndrome and digital eye strain [32].

## 4. Discussion

The evidence suggests that media device use may have consequences for the eye’s health, including the risk of pre-myopia, myopia, digital eye strain, and acute acquired comitant esotropia [12,13,14,15,16,17,18,19,20,21,22,23,24,25,26,27,28,29,30,31,32,33,34,35,36,37].

Viewing distance plays a role if we refer to emerging technologies and devices. The recent sharp increase in the number of children and adolescents spending long periods looking at screens, as well as the availability of technology on laptops or small devices such as pocket-size devices, represent risk factors for eye health [18,21,22,31,33,36]. The literature supports the evidence that media device viewing duration and close distance relate to the risk of myopia, dry eye disease, and acute acquired comitant esotropia [12,14,31,32,35,37]. Small screens and the font size of mobile devices promote a closer viewing distance, leading to ocular fatigue and placing a greater demand on accommodation and vergence than computer screens or printed materials. A reduced blink rate and faster evaporation of the tear film in the case of prolonged media device use may lead to dry eye disease.

AACE in children, especially related to the increased use of electronic devices, may be linked to latent hyperopia and the development of accommodative esotropia. Low fusional divergence, along with increased accommodation and convergence demands from near work, can contribute to the development of esotropia in children with latent hyperopia. Children with low fusional divergence have a reduced ability to relax their convergence and allow the eyes to diverge effectively when looking at distant objects. This can lead to a situation where the eyes remain excessively converged even when they should not be, contributing to strabismus. In children with latent (or uncorrected) hyperopia, the extra effort needed to focus on near objects can trigger excessive convergence. This is further amplified by extended near work, reinforcing the inward deviation of the eyes. Children with weak divergence ability (low fusional divergence) are particularly susceptible to developing esotropia in such circumstances. Essentially, the eye is stuck in an overly converged state because the child cannot easily relax their convergence to compensate for the latent hyperopia, and the demands of near work keep triggering excessive accommodation and convergence. Cycloplegic refraction and measurements of fusional reserves were taken into consideration in the analyzed studies [35,37]. Cycloplegic refraction eliminates the influence of accommodation, providing a more accurate assessment of the child’s refractive error. It helps determine if optical correction of hyperopia will improve the esotropia. Measuring fusional reserves assesses the dynamic component of the child’s ocular alignment. It can help identify any other underlying vergence issues that may require different treatment strategies.

The small number of studies limits the interpretation of the evidence according to different types of mobile media devices used, even if smartphones and other mobile media devices have been frequently associated with ocular problems [35,36].

Considering different age groups, we identified evidence in childhood and in adolescence in many cases. In children aged less than 12 years, the evidence suggests an increased risk of poor eye health after the COVID-19 pandemic due to an increased use of media devices likely correlated with an increased time spent on close-distance activities and digital devices [12,28].

Public health measures should be adopted to avoid the consolidation of these unhealthy behaviors and promote, on the contrary, correct lifestyles. Modifiable factors in the case of excessive screen viewing time include mobile phone use before bedtime, parents’ perception about the child’s habituation to screen time, having a device located in the bedroom, morning mobile screen viewing time, and lack of outdoor play or reading [22].

Lifestyle, including low outdoor activity, Westernized dietary habits, and urban setting, should be considered when referring to risk factors for myopia and dry eye syndrome in minors exposed to media devices [12,15,16,18,21,31].

In line with the Italian Society’s recommendations, we suggest that families’ education about the correct use of media devices is fundamental to protecting minors’ sight. Parents should be educated regarding media device exposure in childhood, modulating it based on clinical evidence and scientific recommendation, limiting especially the use at a close distance. There should be no media device use in children less than 2 years of age, during meals, before bedtime, or as a limited pacifier. Children should use media devices in the presence of a caregiver to supervise both contents and physical posture [6].

Regarding adolescents, the literature reveals an association between the high use of screen devices, reduced levels of physical activity, and the risk of myopia [17]. So, as well as in childhood, sports and physical activity should be promoted among adolescents. In adolescence, as well, rules and limits should be discussed to prevent ocular problems because the increased use of smartphones may result in problems such as dry eye disease, eye irritation, decreased visual acuity, and macular degeneration [17,32].

The connection between myopia and outdoor activity has become a significant area of research in recent years [39,40,41,42,43]. The evidence suggests that spending less time outdoors is a key risk factor for the development and progression of myopia [40]. On the contrary, exposure to natural light may play a protective role. Studies have shown that children who spend more time outdoors, where they are exposed to brighter light, tend to have a lower risk of myopia compared to those who spend more time indoors. The main potential mechanisms involved in the protective effect of time spent outdoors against myopia include exposure to elevated levels and shorter wavelengths of light (daylight), and increased dopamine and vitamin D levels. Vitamin D synthesis, as well as the concentration and release of dopamine influenced by outdoor time, may play a role in inhibiting myopic development, thereby promoting eye growth [40,41,42,43]. So, interventions should be aimed at increasing daily light exposure and outdoor activities [42,43]. Spending time outdoors may also encourage activities like focusing on distant objects, which may help eye health, even if the relationship with myopia remains controversial [40]. In a cross-sectional study of children, more time spent in outdoor sport and activity was associated with lower myopia risk, but there was no similar effect for indoor sports and activity. This suggests that spending time outdoors, rather than physical activity, was protective for eye health [43].

A simple but scientific dialogue with families on media device use should be undertaken during check controls. The evidence suggests that the parents with higher levels of risk perception and more parental mediation were more likely to mediate their child’s eye care behavior. Moreover, minors whose parents had a lower mediation and spent a prolonged period online were at higher risk for a prolonged SVT [43].

Pediatric ophthalmologists are now constantly facing disorders related to the use of electronic devices, particularly if they are used at a close distance. So, the level of parents’ awareness of the potential risks should be considered, and the short and long-term consequences of the incorrect use of the devices should be explained [4,5,10]. Then, recommendations to contain the problem should be shared, including adequate lighting, avoiding prolonged use and display use at night, the distance from the device, and limiting near-use for text and message-sharing activities [5,10]. In the case of reduced visual acuity and without refractive errors in cycloplegia or ocular pathologies, a wash-out period from the devices must be recommended, followed by a follow-up control. In case of concomitant refractive error, an ophthalmologic correction is required. In case of diplopia, orthoptic and neurological evaluations are prescribed to avoid other possible concomitant systemic pathologies.

A limitation of this research is that the reviewed studies were conducted among different populations of different ages, so the results are not generalizable to all of the pediatric population. Further studies are required to better define the risks according to age groups, ethnicity, demographic, and social characteristics in order to strengthened the evidence regarding media device effects on eye health.

## 5. Conclusions

Pre-myopia, myopia, digital eye strain, and acute acquired comitant esotropia are potential time-related short- and long-term outcomes associated with incorrect use of media devices among children and adolescents. Raising awareness among parents and policymakers should be considered key to mitigating and avoiding consequences for children’s and adolescents’ sight. We suggest allocating time during a pediatric routine healthcare visit and promptly referring minors to ophthalmologists in case of ocular symptoms.

## Figures and Tables

**Table 1:** Media device and myopia.

Domain	Author	Country	Age	Main Findings
Myopia	Trovato Battagliola E, 2023 [12]	Italy (Europe)	5–12 years	Out of 803 children, statistically significant decrease in the mean spherical equivalent refraction (*p* = 0.005) and an increase (*p* = 0.016) in the percentage of myopes after COVID-19 confinement were described. The percentage of hyperopes decreased (*p* = 0.001) compared to previous years. Children aged 8–12 years were the most severely affected.
Myopia	Foreman J 2021 [14]	Australia	3–16 years	Screen exposure was associated with increased risks of developing myopia.
Myopia	Wong CWW 2021 [15]	China (Asia)	Less than 18 years	Increased digital screen time and limited outdoor activities were found to be associated with the onset and progression of myopia, aggravated during and beyond the COVID-19 pandemic outbreak period.
Myopia	Harrington SC, 2019 [16]	Ireland (Europe)	6–7 and 12–13 years	Out of 1626 participants, myopia prevalence was higher in adolescents aged 12–13 years and in the case of screen use for more than hours a day (*p* < 0.001).
Myopia	Hansen MH, 2019 [17]	Belgium (Europe)	16–17 years	Out of 1435 participants, the use of screen devices >6 h/day was associated with increased OR for myopia compared with screen device use <2 h/day in both weekdays (OR = 1.95, CI 95% 1.16–3.30, *p* = 0.012) and weekends (OR = 2.10, CI 95% 1.17–3.77, *p* = 0.013).
Myopia	Enthoven CA, 2020 [18]	Netherlands (Europe)	3–9 years	Out of 5.074 children, computer time use correlates with myopia (OR = 1.005, 95% CI = 1.001–1.009).
Myopia	Harrington S, 2023 [19]	Ireland (Europe)	6–7 years	Out of 723 students, a daily screen time of more than 2 h correlates with myopia (SER < 0.5) and pre-myopia (SER > −0.50 D to ≤+0.75 D). It is associated with increased myopic spherical equivalent (*p* < 0.001)], refractive astigmatism (*p* = 0.01), axial length/corneal radius (*p* < 0.001), and a reduced corneal radius (*p* = 0.02).
Myopia	Liu S, 2019 [20]	China (Asia)	6–14 years	Out of 566 participants, a more myopic spherical equivalent refraction and a longer axial length were associated with more time on smartphones and computers, but not on tablets and television. The spherical equivalent refraction decreased by 0.28 D (*p* = 0.042) and 0.33 D (*p* = 0.018) for each 1 h increase in the time spent using smartphones and computers, respectively. A longer axial length was associated with more time spent using smartphones (*p* = 0.006) and computers (*p* = 0.002).
Myopia	Terasaki H, 2017 [21]	Japan (Asia)	8–9 years	Out of 122 students, the duration of computer and smartphone use was significantly correlated with longer axial length (r = 0.24, *p* = 0.008).
Myopia	Guan H, 2019 [22]	China (Asia)	9–11 years	In a sample size of 19.934 students, a greater refractive error links to more than 1 h a day on a computer (−0.025 LogMAR units, *p* = 0.011) and on a smartphone (−0.041 LogMAR units, *p* = 0.001), but not with television.
Myopia	Liu J, 2021 [23]	China (Asia)	<18 years	Out of 3831 adolescents during the COVID-19 pandemic, any 1 h increase in daily digital screen use correlates with 1.26 OR [odds ratio] higher risks of myopic progression (*p* < 0.001). Smartphones and computers are associated with higher risks of myopic progression than television (OR = 2.02 and OR = 1.813, respectively)
Myopia	Saxena R, 2015 [24]	India (Asia)	5–15 years	Out of 9884 children, those watching television for more than 2 h a day and playing games by computer, video, or mobile devices were more at risk of myopia (*p* < 0.001). Myopia was more likely linked with studying in private schools rather than government ones.
Myopia	Singh NK, 2019 [25]	India (Asia)	5–15 years	In a sample size of 1234 children, myopia was more frequent in the case of those involved in games using computer, video, or mobile devices for more than 2 h a day (*p* < 0.001).
Myopia	Yang GY, 2020 [26]	China (Asia)	2–7 years	Out of 26,433 preschoolers, a statistically significant association was observed between initial screen exposure at 0–1 year old and myopia (adjusted PR 95% CI was 3.81 (2.00, 7.26).
Myopia	Chang *p*, 2021 [27]	China (Asia)	6–17 years	29,719 participants. After the COVID-19 outbreak, spherical equivalent refraction significantly (*p* < 0.001) decreased; a significant increase in high myopia was found (*p* < 0.001).
Myopia	Alvarez Peregrina C, 2021 [28]	Spain (Europe)	5–7 years	During COVID-19 home confinement, there was a significant decrease in SER (*p* ≤ 0.001).
Myopia	Wang W, 2021 [29]	China (Asia)	6–18 years	Out of 3461 participants, during the COVID-19 period, the mean spherical equivalent refraction worsened compared to the previous year (*p* <0.001), mostly in the case of computer (−2.03 ± 2.37 D, *p* = 0.0017) and cell phone (−2.02 ± 2.09 D, *p* = 0.0028) use for online courses rather than television (−1.10 ± 1.49 D).
Myopia	Mc Crann S, 2020 [30]	Ireland (Europe)	10–18 years	217 students (87 aged 10 to 12 years and 130 12 to 18 years) had an increased myopia risk correlated with smartphone usage. Myopic refractive error was significantly associated with increasing daily usage.

Legend: OR: odd’s ratio CI: confidence interval.

**Table 2:** Digital eye strain and media device.

Domain	Author	Country	Age	Main Findings
Digital eye strain	Bhattacharya S, 2022 [13]	India (Asia)	Less than 18 years old	Digital eye strain increased during the COVID-19 pandemic and correlates to the effect of digital screen on eyes
Digital eye strain	Moon JH, 2016 [31]	Korea (Asia)	7–12 years	In a sample size of 916 children, smartphone and computer use correlates to the prevalence of dry eye (*p* < 0.001). After the cessation of smartphone use for 4 weeks, both subjective symptoms and objective signs of the disease improved. Increased outdoor activity time reduced the rate of DES. The rate of DES was higher in urban than in rural children.
Digital eye strain	Kim J, 2016 [32]	Korea (Asia)	Mean age 15 years	Out of 715 adolescents, higher prevalence rates for ocular symptoms were observed in those with greater exposure to smartphones, in particular inflammation (excessive/persistent use OR 1.88, 95% CI 1.12–3.16), lacrimation (excessive/intermittent use OR 1.96, 95% CI 1.22–3.14; excessive/persistent use OR 2.12, 95% CI 1.31–3.46), redness (OR 2.05, 95% CI 1.24–3.38), and lacrimation (intermediate exposure OR 1.92, 95% CI 1.21–3.05; higher exposure OR 1.95, 95% CI 1.22–3.12; extreme exposure OR 2.49, 95% CI 1.54–4.03). Prolonged daily smartphone use is linked with a higher risk of multiple ocular symptoms.
Digital eye strain	Mohan A, 2021 [33]	India (Asia)	13 ± 2.45 years (range 10–18)	In the COVID-19 era, among 217 participants, the mean duration of the use of digital devices was 3.9 ± 1.9 h, which was higher compared to the pre-pandemic period (1.9 ± 1.1 h, *p* = <0.0001); 36.9% used digital screens for more than 5 h as compared to 1.8% before the pandemic. Prevalence of DES was 50.23% (109/217). Multivariate analysis revealed that the use of a smartphone (*p* = 0.003), of a device longer than 5 h a day (*p* = 0.0007), and of mobile games longer than 1 h a day (*p* = 0.0001) should be considered risk factors.
Digital eye strain	Mohan A, 2021 [34]	India (Asia)	14.47 ± 1.95 years (range 10–17)	In the pandemic era, online lessons for more than 4 h a day represented a risk factor for the symptomatic convergence insufficiency symptoms, including exophoria, negative fusional vergence, negative relative accommodation, and accommodation amplitude.
Digital eye strain	Mohan A, 2021 [35]	India (Asia)	12.5 ± 4.2 years	During COVID-19, students with acute acquired comitant esotropia were attending online classes > 4 h per day on smartphones having an average size of 5.5 inches.
Digital eye strain	Rechichi C, 2017 [36]	Italy (Europe)	6.9 ± 2 years	Asthenopia (especially headache, eyelid tic, transient diplopia, and dizziness), heterophoria (22.5%), ametropic eyes (90.4%), astigmatism (58.5%), and absence of fine stereopsis, were statistically more frequent in children playing video games.

Legend: OR: odds ratio CI: confidence interval.

**Table 3:** Acute acquired comitant esotropia and media device.

Domain	Author	Country	Age	Main Findings
Acute acquired comitant esotropia	Mohan A, 2021 [35]	India	12.5 ± 4.2 years (range 6–18 years)	During COVID-19, students with acute acquired comitant esotropia attended online classes > 4 h per day on smartphones having an average size of 5.5 inches.
Acute acquired comitant esotropia	Hyo Seok Lee, 2016 [37]	South Korea	13.33 ± 3.31 years (range 7–16 years)	Children with smartphone use of more than 4 h daily in 7 months were at high risk for acute acquired comitant esotropia.

## Data Availability

Not applicable.

## Abbreviations

SVT	Screen viewing time
DES	Digital eye strain
AACE	Acute acquired comitant esotropia
SER	myopic spherical equivalent refraction
AL	axial length
